# Metabolic Determinants of PCSK9 Regulation in Women with Polycystic Ovary Syndrome: The Role of Insulin Resistance, Obesity, and Tobacco Smoke Exposure

**DOI:** 10.3390/ijms27010331

**Published:** 2025-12-28

**Authors:** Justyna Niepsuj, Agnieszka Piwowar, Grzegorz Franik, Anna Bizoń

**Affiliations:** 1 Department of Toxicology, Faculty of Pharmacy, Wroclaw Medical University, 50-556 Wroclaw, Poland; 2 Department of Endocrinological Gynecology, Medical University of Silesia, 40-752 Katowice, Poland

**Keywords:** polycystic ovary syndrome, PCSK9, metabolic abnormalities, endocrine disorders, smoking

## Abstract

The aim of this study was to examine associations involving serum proprotein convertase subtilisin/kexin type 9 (PCSK9) in metabolic disturbances observed in women with polycystic ovary syndrome (PCOS), with particular emphasis on the potential impact of tobacco smoke exposure. The study included 88 women: 60 with PCOS (23 smokers and 37 non-smokers) and 28 without PCOS. Selected biochemical and molecular biomarkers related to lipid metabolism, oxidative stress, and inflammation were assessed. No significant differences in PCSK9 levels were observed among non-smoking women with PCOS, smoking women with PCOS, and non-smoking women without PCOS. However, in women with PCOS, excess body weight and insulin resistance were associated with increased PCSK9 concentrations. Significant correlations between PCSK9, lipid profile parameters, and the Castelli and triglycerides-glucose indices suggest a potential role of PCSK9 as a biomarker of dyslipidemia and cardiometabolic risk. Elevated PCSK9 levels may contribute not only to increased low-density lipoprotein cholesterol but also to enhanced formation of oxidized low-density lipoprotein, which is particularly detrimental to cardiovascular and metabolic health. Vitamin D levels were more strongly associated with smoking status and insulin resistance than with excess body weight. Overall, these findings indicate that PCSK9 regulation in PCOS may be driven predominantly by metabolic factors rather than PCOS status or smoking per se, and that metabolic status and vitamin D deficiency should be considered when assessing cardiometabolic risk in this population.

## 1. Introduction

Polycystic ovary syndrome (PCOS) is one of the most common endocrine disorders affecting women of reproductive age. According to the Rotterdam Criteria, a diagnosis of PCOS is established when at least two of the fallowing three features are present: oligo- and/or anovulation, clinical and/or biochemical signs of hyperandrogenism, and polycystic ovarian morphology (PCOM) [1,2]. However, these diagnostic criteria do not fully capture the broad spectrum of metabolic and endocrine abnormalities associated with the disorder. Women with PCOS are at an increased risk of developing obesity, dyslipidemia, insulin resistance (IR), type 2 diabetes (T2D), and cardiovascular disease (CVD) [3].

Although the precise pathogenesis of PCOS remains unclear, accumulating evidence indicates that both genetic predisposition and environmental factors contribute to its development [4]. Because cholesterol (CHO) serves as a precursor for sex steroid synthesis, disturbances in lipid metabolism may represent not only a consequence but also a potential contributing factor to PCOS pathophysiology [5]. Our previous studies demonstrated that women with PCOS and hyperlipidemia exhibit significantly higher levels of free and total testosterone, androstenedione, and an increased free androgen index (FAI). Moreover, the concentration of sex hormone-binding globulin, which regulates the bioavailability of androgens to target tissues, was found to be negatively correlated with high-density lipoprotein cholesterol (HDL-C) levels [6,7]. These findings suggest that alterations in cholesterol metabolism may influence circulating sex hormones concentrations and thereby contribute to the onset and progression of PCOS.

Proprotein convertase subtilisin/kexin type 9 (PCSK9) is a key regulator of lipid metabolism that promotes the degradation of low-density lipoprotein receptor (LDLR), thereby modulating circulating low density lipoprotein cholesterol (LDL-C) levels [8]. PCSK9 is predominantly expressed in hepatocytes, where it facilitates LDLR internalization and lysosomal degradation [9]. Increasing evidence suggests that dysregulation of the PCSK9-LDLR pathway may contribute to the metabolic abnormalities observed in PCOS. In particular, Mondal et al. demonstrated that hyperandrogenemia and hyperhomocysteinemia, two common features in PCOS, share a PCSK9-LDLR-dependent mechanism that disrupts cholesterol homeostasis, leading to impaired lipid clearance and exacerbated dyslipidemia [10]. Their findings support the hypothesis that altered PCSK9 activity may play a mechanistic role not only in lipid disturbances but also in the broader metabolic dysfunction characteristic of PCOS.

Further support for the involvement of PCSK9 in female metabolic regulation comes from experimental studies. Yang et al. demonstrated that PCSK9 participates in lipid metabolism disorders induced by estrogen deficiency and that estrogen regulates PCSK9 and LDLR at the post-transcriptional level, underscoring the importance of PCSK9 in female hormonal homeostasis [11]. Moreover, PCSK9 expression is modulated by IR, with insulin shown to upregulate PCSK9 expression and severe IR associated with elevated circulating PCSK9 levels [12]. These observations suggest that PCSK9 may integrate multiple metabolic signals relevant to PCOS pathophysiology. Consequently, assessing PCSK9 concentrations in women with PCOS may provide valuable insights into the interplay between dyslipidemia, IR, androgen excess, and related metabolic disturbances.

Oxidative stress (OS) is another crucial factor implicated in the pathogenesis of PCOS. It is defined as an imbalance between the production of reactive oxygen species (ROS) and the antioxidant defense mechanisms responsible for their neutralization [13]. In PCOS, OS may contribute to disease progression through several interrelated mechanisms. It can impair insulin signaling, thereby promoting IR [14]. In turn, both IR and dyslipidemia may further enhance ROS generation through endoplasmic reticulum stress and lipid peroxidation. Additionally, OS may activate redox-sensitive transcription factors involved in systemic inflammation, amplifying lipid peroxidation and exacerbating metabolic dysregulation and clinical manifestations of PCOS [15,16].

Cigarette smoking represents an additional environmental factor that may aggravate PCOS manifestations. A study conducted in China demonstrated that women with PCOS who smoke exhibit significantly higher testosterone levels and more severe depressive symptoms [17]. Smoking has also been shown to promote IR, increase the risk of T2D, and serve as a source of both exogenous and endogenous ROS generation [18]. Therefore, smoking status should be considered an important confounding and potentially modifying factor in studies evaluating metabolic and oxidative disturbances in PCOS. To comprehensively assess OS, not only total oxidant status (TOS) was evaluated, but also oxidized-LDL (ox-LDL) as a marker of lipid peroxidation, along with the antioxidant capacity (AC) and paraoxonase 1, 2, and 3 (PON1-3) levels. PON1 and PON3 are HDL-associated enzymes that exert antioxidant and anti-atherogenic effects by preventing lipid oxidation, whereas PON2 is an intracellular enzyme expressed in various tissues, including vascular cells, where it mitigates intracellular OS [19]. Combined with analyses of PCSK9, LDLR, lipid profile, hormonal indices, and nicotine exposure, these OS markers provide a comprehensive overview of the interconnected physiological processes underlying PCOS.

Therefore, the primary objective of this study was to evaluate the role of serum PCSK9 in the metabolic and pathophysiological disturbances associated with PCOS. We hypothesized that serum PCSK9 concentrations are associated with lipid and glucose metabolism disturbances in women with PCOS, and that these associations may be modified by smoking and vitamin D deficiency. Therefore, we aimed to investigate the relationships between PCSK9 concentrations and lipid metabolism parameters, IR, OS markers, and androgen-related metabolic alterations. In addition, we sought to assess the potential modifying effects of cigarette smoking and vitamin D status on these associations, to better elucidate their contributions to cardiometabolic risk in women with PCOS.

## 2. Results

### 2.1. Comparison of the Control Group with Non-Smoking and Smoking Women with PCOS

A comparison of non-smoking and smoking women with PCOS with control group is presented in Table 1. Significant differences were observed among the three groups with respect to LDLR concentrations, oxLDL, PON1, vitamin D, and CRP, as well as HOMA-IR values, Castelli indices I and II, and the TyG index. Smoking women with PCOS exhibited the highest LDLR and CRP concentrations and the lowest vitamin D levels. Moreover, this group demonstrated the highest value of HOMA-IR, TyG, and Castelli indices I and II. No significant differences in PCSK9 concentrations were observed between non-smoking and smoking women with PCOS and non-smoking controls.

### 2.2. Analysis of the Studied Parameters in PCOS Women Grouped by BMI and HOMA-IR

Stratification of women with PCOS based on BMI (<25.0 vs. ≥25.0) revealed significant differences in PCSK9, vitamin D and CRP concentrations, as well as in Castelli indices I and II and the TyG index. In addition, participants with BMI ≥ 25.0 exhibited significantly altered PON2 and PON3 concentrations compared with normal-weight women.

Similarly, stratification according to insulin resistance (HOMA-IR < 2.0 vs. ≥2.0) demonstrated significant differences in PCSK9 and CRP concentrations, Castelli indices I and II, and the TyG index. Women with HOMA-IR < 2.0 presented significantly higher vitamin D levels but lower PON1 concentrations compared with those with HOMA-IR ≥ 2.0. These results are summarized in Table 2.

### 2.3. Analysis of the Studied Parameters in Smoking vs. Non-Smoking PCOS Women, Stratified by BMI

Further subgroups analyses were performed separately in non-smoking and smoking women with PCOS stratified by BMI (Table 3). In both subgroups, overweight or obesity was associated with significantly increased values of Castelli indices I and II, the TyG index, and approximately two-fold higher CRP concentrations. Alterations in PON2 and PON3 concentrations were observed exclusively in non-smoking women with PCOS.

### 2.4. Analysis of the Studied Parameters in Smoking vs. Non-Smoking PCOS Women, Stratified by HOMA-IR

Analogous analyses stratified by insulin resistance status within smoking and non-smoking PCOS subgroups revealed significant differences in Castelli indices I and II, the TyG index, and CRP concentrations in both subgroups. Additionally, in the non-smoking subgroup, significant changes were noted in PCSK-9 and vitamin D concentrations (Table 4).

### 2.5. Correlations

Correlation analyses conducted in the entire group of women with PCOS are presented in Table 5. Significant associations were observed between PCSK9, LDLR, oxLDL, the TyG index, vitamin D, PON1 concentrations, and evaluated parameters. No significant correlations were detected for PON2 and PON3; therefore, these parameters were excluded from further analyses.

Correlation coefficients were additionally analyzed in smoking women with PCOS (Table 6). Similarly, no significant correlations were observed with PON2 or PON3 concentrations.

Additional correlation analyses were performed in the entire group of women with PCOS as well as in the subgroup of smoking women with PCOS. Associations were assessed between PCSK9, LDLR, oxLDL, the TyG index, vitamin D, PON1 concentrations, selected sex hormones, sex hormone-binding globulin (SHBG), the free androgen index (FAI), glucose levels (fasting and post-oral glucose tolerance test), and insulin concentrations. The results of these analyses are provided in Tables S1 and S2 of the Supplementary Materials.

## 3. Discussion

Disturbances in lipid metabolism are well established as a key component of the pathophysiology of PCOS [20]. Cholesterol serves as the biochemical precursor of all steroid hormones as well as vitamin D3 (via 7-dehydrocholesterol in the skin) and may therefore influence hormonal balance. Women with PCOS frequently present with lipid abnormalities which, in combination with IR and other metabolic disturbances, contribute to an increased risk of cardiovascular disease [21,22,23]. Importantly, dyslipidemia in PCOS has been reported to occur independently of BMI [23].

Given the role of PCSK9 in mediating LDLR degradation and regulating LDL-C levels, the present study aimed to evaluate PCSK9 concentrations in non-smoking and smoking women with PCOS in the context of excess body weight and insulin resistance. No significant differences in PCSK9 levels were observed among non-smoking and smoking women with PCOS and non-smoking women without PCOS, suggesting that PCSK9 alterations may not be directly attributable to PCOS per se but rather to accompanying metabolic and environmental factors.

Previous studies have demonstrated that circulating PCSK9 concentrations are higher in females than in males and can be regulated by hormones such as insulin and glucagon, underscoring the sensitivity of this protein to endocrine and metabolic regulation [24]. Furthermore, Filippatos et al. [25] and Tóth et al. [26] reported significantly higher PCSK9 levels in obese individuals compared with normal-weight controls, supporting the concept that PCSK9 may act as a metabolic biomarker linking excess adiposity with lipid dysregulation and increased cardiovascular disease risk.

Evidence regarding PCSK9 concentrations specifically in PCOS remains inconsistent. Our previously findings demonstrated higher serum PCSK9 levels in women with PCOS compared to age and BMI-matched healthy controls, suggesting a possible contribution of PCSK9 to the metabolic disturbances associated with the syndrome [27]. In contrast, Xavier et al. reported that PCSK9 concentrations were not independently elevated in PCOS after accounting for metabolic status; however, associations with lipid and androgen metabolism were observed, indirectly implicating PCSK9 in PCOS pathophysiology [28]. These results are further supported by an experimental study conducted in a PCOS mouse model [29], which demonstrated that a high-fat diet may play an important role in inducing abnormally increased PCSK9 expression. Collectively, these findings suggest that PCSK9 dysregulation in PCOS may be driven primarily by metabolic and hormonal abnormalities rather than by the syndrome itself.

Although we did not observe significant differences in PCSK9 levels with tobacco smoke exposure, smoking is well-recognized inducer of OS and systemic inflammation, both of which can disrupt cholesterol homeostasis and alter intracellular signaling pathways involved in lipid metabolism [30,31,32].

In present study, tobacco exposure was objectively assessed using cotinine concentrations, however, dose–response analyses could not be evaluated, representing a limitation of the analysis.

Taking together, these findings highlight the multifactorial regulation of PCSK9 in PCOS, involving not only intrinsic metabolic and endocrine disturbances, but also environmental factors such as tobacco smoke exposure. The interplay among OS, dyslipidemia, IR, hormonal imbalance, and external exposures may collectively shape PCSK9 expression patterns in affected women.

Consistent with this concept, smoking women with PCOS exhibited the highest CRP levels and the lowest total antioxidant capacity, indicating the presence of low-grade inflammation and impaired antioxidant defense. These conditions may promote enhanced activation of sterol regulatory element-binding protein 2 (SREBP2) and hepatocyte nuclear factor-1 alpha (HNF1α) dependent transcription of PCSK9, thereby linking tobacco exposure to impaired lipid metabolism and increased cardiovascular disease risk [33]. This regulatory pathway was also proposed in study by Guo et al. [29], who suggested that a high fat diet may play an important role in inducing abnormally increased PCSK9 expression via SREBP2 upregulation.

In this context, significant positive correlations were observed between PCSK9 levels and total cholesterol, LDL-C, oxLDL, and Castelli indices in both study groups, with stronger associations in the smoking women with PCOS. These relationships further support the role of PCSK9 in lipid dysregulation and atherogenic risk.

Stratification by BMI and HOMA-IR also revealed significantly higher PCSK9 concentrations in women with BMI ≥ 25.0 and HOMA-IR ≥ 2.0 compared with their respective reference groups. These findings suggest that both excess body weight and IR are important determinants of elevated PCSK9 levels. In line with these observations, Ruscica et al. [34] reported positive correlations between PCSK9 and insulin, glucose, HOMA-IR in a 17-year follow-up study. Insulin resistance has been identified as a key regulator of PCSK9 expression, providing a mechanistic link between disturbances in glucose metabolism and lipid homeostasis. Additional clinical studies have demonstrated elevated PCSK9 levels in patients with type 2 diabetes and metabolic syndrome, even after adjustment for LDL-C levels. Similarly, Levenson et al. [35] observed increased PCSK9 levels even in young females with T2D and metabolic dysfunction. Moreover, elevated PCSK9 has also been proposed as a prognostic biomarker of cardiovascular events, particularly in women with T2D [34].

In the present study, women with HOMA-IR ≥ 2.0 exhibited higher PCSK9 concentrations not only in the overall PCOS group but also within the non-smoking subgroup, underscoring the strong impact of IR on PCSK9 regulation irrespective of tobacco exposure. Correlation analyses revealed significant positive associations between PCSK9 and total cholesterol, LDL-C, triglycerides, follicle-stimulating hormone (FSH), and anti-Müllerian hormone (AMH) levels.

Castelli indices I and II, which integrate lipid parameters to improve cardiovascular risk prediction, were significantly influenced by PCOS status, tobacco smoke exposure, BMI, and IR. Although these indices rely solely on lipid parameters, the TyG index incorporates both triglyceride concentrations and glucose levels and is considered a surrogate marker of IR. Given that approximately 30–40% of women with PCOS exhibit impaired glucose metabolism, IR, or T2D [36,37,38], the TyG index has been proposed as a useful tool to distinguish metabolic from non-metabolic PCOS phenotypes [39,40,41]. In the present study, TyG values, similarly to the Castelli indices, were influenced by PCOS status, smoking exposure, BMI and HOMA-IR. Notably, stronger correlations between TyG and lipid and hormonal parameters were observed in smoking women with PCOS, with triglycerides showing an exceptionally strong association with TyG (R = 0.98), suggesting that tobacco smoke exposure may exacerbate dyslipidemia-related metabolic disturbances. Furthermore, a positive correlation between TyG and PCSK9 concentrations was noted in both study groups, supporting the emerging link between PCSK9, lipid abnormalities, and glucose-insulin homeostasis.

Vitamin D represents another important modulator of lipid metabolism and hormonal homeostasis. Vitamin D regulates estrogen and androgen synthesis through modulation of aromatase activity, supports ovulation, and influences luteinizing hormone (LH) and FSH receptor expression, and AMH signaling in PCOS [42,43,44]. Vitamin D deficiency (<20 ng/mL) has been associated with dyslipidemia, increased CVD risk, and elevated atherogenic indices, including Castelli ratios [45]. Inverse association between vitamin D concentrations and IR has also been consistently revealed [46].

In present study, vitamin D concentrations were generally low, with many participants exhibiting levels below 12 ng/mL. According to the Polish Guidelines for Vitamin D Deficiency, optimal concentrations range between 30–50 ng/mL (75–125 nmol/L), with even higher values recommended for women planning pregnancy. Significant differences in vitamin D status were observed between smoking and non-smoking women with PCOS and controls, as well as in PCOS subgroups stratified by HOMA-IR but not by BMI. These findings suggest that IR and tobacco smoke exposure, rather than excess body weight, may be key determinants of vitamin D deficiency in PCOS. Vitamin D levels were inversely correlated with insulin and HOMA-IR, and women with lower vitamin D levels exhibited higher PCSK9 concentrations, suggesting a potential link between vitamin D deficiency and metabolic dysregulation. Experimental studies support the regulatory role of vitamin D in oxidative and atherogenic pathways, including downregulation of PCSK9 expression and enhancement of LDLR activity. Although trends consistent with these mechanisms were observed, correlations between vitamin D and PCSK9 did not reach statistical significance.

Markers of oxidative stress were markedly altered in women with PCOS, particularly among smokers and those with excess body weight. Reduced PON concentration and elevated oxLDL levels support the hypothesis that oxidative imbalance plays a pivotal role in PCOS pathophysiology. Increased generation of reactive oxygen species (ROS) promotes lipid peroxidation, leading to the accumulation of oxLDL, which is closely associated with IR, dyslipidemia, and obesity [47,48]. Moreover, PCSK9 has been shown to promote LDL oxidation (oxLDL) and impair endothelial antioxidant defenses, thereby contributing to vascular oxidative injury and atherogenesis [49].

In conclusion, the present findings indicate that PCSK9 regulation in PCOS is influenced not only by the syndrome itself but also by metabolic status, IR, and excess body weight. Significant correlations between PCSK9 and total lipid, oxidative, and cardiovascular indices support its potential role as a biomarker of cardiometabolic risk in this population. Collectively, these results suggest that therapeutic strategies targeting PCSK9, correcting vitamin D deficiency, and promoting smoking cessation may provide clinically meaningful benefits for improving cardiometabolic and reproductive outcomes in women with PCOS.

## 4. Materials and Methods

The study was conducted in a group of 88 women who were hospitalized between 14 July 2022 and 10 November 2022 at the Gynecological Endocrinology Clinic of the Medical University of Silesia in Katowice, Poland. A total of 60 of them were diagnosed with PCOS according to Rotterdam Criteria, which indicates that PCOS may be confirmed when 2 of 3 following symptoms are present: biochemical and or clinical hyperandrogenism, ovulatory dysfunction (amenorrhea or menstrual cycles longer than 35 days), and polycystic ovaries on ultrasound [1]. Among this group, 23 women were identified as smokers and 37 as non-smoking. Information on tobacco smoke exposure was obtained from personal interviews and verified by measurement of serum cotinine level, the main metabolite of nicotine, using a commercial ELISA test (Cotinine ELISA, Cat. No. CO096D, Calbiotech Inc., El Cajon, CA, USA).

Additionally, 28 women in case of which diagnosis of PCOS was excluded took part in the study as control group. Women in the control group were admitted due to menstrual irregularities requiring diagnostic evaluation. PCOS was excluded in all control subjects based on a comprehensive clinical assessment, including a detailed menstrual history, evaluation of biochemical and clinical hyperandrogenism (including assessment of hirsutism), and pelvic ultrasonography for the presence of PCOM. None of the women examined at the Medical University of Silesia met the diagnostic criteria for PCOS, and their hormonal profiles were within normal reference ranges. Moreover, women meeting the following exclusion criteria were excluded: alcohol abuse, diabetes, Cushing’s syndrome, adrenal tumor, and hypertension. The study was approved by Bioethical Committee of the Wroclaw Medical University (KBN No. 254/2021).

Blood samples were collected under standardized conditions, centrifuged, aliquoted, and stored at −80 °C. Serum samples were transported on dry ice by a certified courier service and stored at −80 °C until analysis.

### 4.1. Clinical Laboratory Parameters

During hospitalization, blood samples were collected during follicular phase (between 3 and 5 days of the menstrual cycle). Numerous parameters (glucose, insulin, LDL-C, HDL-C, CHO, triglycerides (Ty), free and total testosterone, androstenedione, dehydroepiandrosterone sulfate, luteinizing hormone, follicle-stimulating hormone, Anti-Müllerian Hormone, sex hormone-binding globulin) were published in our previous manuscript [20]. All parameters were measured as part of routine diagnostic procedures, following the methods outlined in our previous publication [21]. During hospitalization, serum 25-hydroxyvitamin D [25(OH)D] levels were measured using the Elecsys Vitamin D Total III assay (Roche Diagnostics, Mannheim, Germany) on a Cobas automated analyzer. The assay employed a competitive electrochemiluminescence immunoassay (ECLIA) method designed for the quantitative determination of total 25(OH)D. The procedure involved the release of 25(OH)D from vitamin D-binding protein, followed by competition between 25(OH)D and a labeled vitamin D reagent for binding sites. The generated electrochemiluminescence signal was inversely proportional to the concentration of 25(OH)D in the sample.

Additionally, Castelli index I (TC/HDL-C), II (LDL-C/HDL-C) and the triglycerides and glucose (TyG) index were calculated.

### 4.2. Biochemical and Molecular Biomarkers Related to Lipid Metabolism, Oxidative Stress, and Inflammation

All biochemical and molecular biomarkers (PCSK9, LDLR, oxLDL, and PONs) were measured in serum.

PCSK9 concentration was measured using Elisa Kit (CUSABIO, Cat. No. CSB-EL017647HU, Houston, TX, USA). The assay employed a competitive enzyme immunoassay method. Samples were added to microtiter plates pre-coated with PCSK9 together with an HRP-conjugated antibody specific to PCSK9. The competitive inhibition reaction occurred between PCSK9 pre-coated on the plate, and those in the samples. Subsequently, a substrate solution was added, resulting in color development that was inversely proportional to the concentration of PCSK9 in the sample. The color intensity was measured using a microplate reader (Synergy HTX Multi-Mode Microplate Reader; BioTek Instruments, Winooski, VT, USA) at 450 nm, with wavelength correction set at 570 nm.

Concentration of LDLR was assayed using Human Low-Density Lipoprotein Receptor, LDLR ELISA Kit (CUSABIO, Cat. No. CSB-E08950h, Houston, TX, USA). The assay was based on a quantitative sandwich enzyme immunoassay technique. An antibody specific to LDLR was bound to the wells, and LDLR present in the samples bound to the antibody. After removing any unbound material, a biotin-conjugated Horseradish Peroxidase (HRP) was added. After a wash, a substrate was added, and color development occurred in proportion to the amount of LDLR bound in the first step. Intensity of the color was determined by using a microplate reader set to 450 nm and with wavelength correction set to 570 nm.

Concentration of ox-LDL was assayed using Elisa Kit (CUSABIO, Cat. No. CSB-E07931h, Houston, TX, USA). The principle of the assay was based on competitive inhibition enzyme immunoassay technique. The plates were pre-coated with oxLDL, and samples were added together with an HRP-conjugated antibody specific to oxLDL. The competitive inhibition reaction occurred between pre-coated oxLDL and oxLDL in the samples. Then a substrate solution was added, color development was inversely proportional to the concentration of oxLDL in the sample. The intensity of the color was measured using a microplate reader at 450 nm with wavelength correction set at 570 nm.

The concentrations of PON1, PON2, PON3 were determined using Elisa Kits (ELK Biotechnology, Cat. No. ELK1925, ELK4072, and ELK3696, respectively; Denver, CO, USA). All assays were based on a sandwich enzyme immunoassay principle. Microtiter plates were pre-coated with antibodies specific to the corresponding PON isoforms. After incubation with samples, avidin conjugated to HRP was added, followed by incubation and washing steps. Next, TMB substrate was added, and color development occurred only in wells containing the specific PON isoforms. The reaction was terminated by the addition of sulfuric acid, and the intensity of the color was measured spectrophotometrically at 450 nm.

AC was assayed using colorimetric test system for the determination of the antioxidative capacity in EDTA plasma and serum (Cat. No. IC5200; ImmuChrom GmbH, Heppenheim, Germany).

TOS was determined using a previously established procedure [22]. In this method, oxidant compounds in the samples converted the ferrous ion–o-dianisidine complex into ferric ions. The reagents used included o-dianisidine dihydrochloride (Cat. No: D3235) and ammonium iron (II) sulfate hexahydrate (Cat. No. 203504), both sourced from Sigma-Aldrich, Taufkirchen, Germany. The resulting ferric ions formed a colored complex with xylenol orange (Cat. No. 398187; Sigma-Aldrich, Taufkirchen, Germany). The intensity of the color, measured spectrophotometrically at 340 nm, was proportional to the total oxidant concentration in the sample.

CRP was measured using High Sensitivity C-Reactive Protein ELISA kit intended for the quantitative determination of CRP in human serum or plasma (Cat. No. CR375C, Calbiotech, Inc., El Cajon, CA, USA). 

### 4.3. Statistical Analysis

All calculations were performed using the Statistica Software Package, version 13.3 (StatSoft, Kraków, Poland, Polish version). We used the Shapiro–Wilk test to assess normality of the variables, and Levene’s test to test the homogeneity of variances. Depending on the results, Student’s *t*-test or the non-parametric U Mann–Whitney test was used to assess differences between subgroups. The comparison among three subgroups was performed using the Kruskal–Wallis one-way analysis of variance by rank. In addition, the Bonferroni post hoc test was conducted after the Kruskal–Wallis test to determine specific intergroup differences not captured by the primary analysis. The values were expressed as mean ± standard deviation, median, 1st quartile, and 3rd quartile. Correlations between parameters were assessed using Spearman’s rank-order correlation coefficient. We considered *p* < 0.05 as statistically significant in all analyses.

## 5. Conclusions

Circulating PCSK9 concentrations did not differ significantly among smoking women with PCOS, non-smoking women with PCOS and non-smoking controls, suggesting that PCSK9 levels are not directly determined by PCOS status or tobacco smoke exposure. Instead, PCSK9 concentrations were more closely associated with metabolic disturbances, particularly excess body weight and insulin resistance. Significant correlations between PCSK9 and atherogenic lipid parameters support its involvement in lipid dysregulation and cardiometabolic risk in women with PCOS. Moreover, insulin resistance and decreased concentration of vitamin D were linked to adverse metabolic profile, while smoking appeared to exacerbate oxidative stress and inflammation rather than directly influencing PCSK9 levels. These findings suggest that metabolic status play a central role in PCSK9 regulation in PCOS and should be considered when assessing cardiometabolic risk in this population.

### Limitations

Several limitations of the present study should be acknowledged. The relatively limited number of participants in both the PCOS and control group may have reduced the statistical power of the analyses. Additionally, heterogeneity in tobacco smoke exposure, reflected by a wide range of self-reported cigarette exposure and cotinine concentrations, without pack-years, may have influenced the observed associations. Furthermore, although women in the control group did not meet the diagnostic criteria for PCOS and exhibited normal hormonal profiles, the presence of menstrual irregularities in some individuals may have introduced additional variability. Therefore, the results of this study should be interpreted with caution and considered exploratory rather than definitive.

## Figures and Tables

**Table 1:** Characteristics and studied parameters in serum of smoking and non-smoking women with PCOS and in non-smoking women without PCOS.

Parameters		Women		
	Non-Smoking Without PCOS (0)	Non-Smoking with PCOS (1)	Smoking with PCOS (2)	*p* Value Between 3 Groups
	*n* = 28	*n* = 37	*n* = 23	
Age (years)	26.71 ± 5.87 27.00 (22.00–31.00)	24.51 ± 4.79 24.50 (22.00–27.00)	26.46 ± 4.47 25.50 (23.00–29.00)	0.324
BMI (kg/m^2^)	22.62 ± 4.58 21.63 (20.57–23.03)	25.15 ± 7.14 23.66 (20.42–29.49)	27.30 ± 7.57 25.55 (21.01–31.25)	0.064
HOMA-IR	1.09 ± 0.52 1.18 (0.70–1.41)	2.07 ± 1.60 1.50 (0.98–2.76)	2.17 ± 2.11 1.54 (1.09–2.41)	0.021 * for 0–2; 0–2
Cotinine (ng/mL)	0.46 ± 0.14 0.41 (0.41–0.41)	0.50 ± 0.34 0.41 (0.41–0.41)	44.72 ± 18.29 78.87 (30.75–60.42)	0.000 * for 0–2; 1–2
Castelli index I	2.69 ± 0.67 2.56 (2.22–2.96)	3.09 ± 0.89 3.01 (2.61–3.33)	3.32 ± 1.19 2.90 (2.39–4.36)	0.002 * for 0–2; 0–2
Castelli index II	1.45 ± 0.57 1.38 (1.12–1.64)	1.70 ± 0.61 1.64 (1.24–1.97)	1.81 ± 0.84 1.58 (1.19–2.22)	0.004 * for 0–1
TyG index	7.99 ± 0.36 7.94 (7.74–8.28)	8.20 ± 0.52 8.03 (7.76–8.53)	8.40 ± 0.53 8.25 (8.01–8.83)	0.045 * for 0–2
LDLR (ng/mL)	14.89 ± 6.72 14.27 (8.87–18.07)	17.65 ± 6.56 16.80 (12.27–21.73)	18.03 ± 10.30 17.33 (9.00–21.40)	0.009 * for 0–1
oxLDL (mU/mL)	35.94 ± 12.22 33.72 (29.25–46.43)	36.79 ± 13.08 37.23 (27.76–45.89)	41.55 ± 12.13 42.92 (34.39–51.85)	0.009 * for 0–2
Vitamin D (ng/mL)	28.52 ± 14.28 28.10 (19.50–32.90)	25.59 ± 10.62 24.20 (18.60–32.40)	23.87 ± 7.66 24.25 (17.60–28.10)	0.027 * for 0–2
PCSK9 (ng/mL)	9.32 ± 3.39 9.82 (7.61–11.59)	8.46 ± 3.47 8.08 (5.98–10.31)	9.34 ± 2.97 8.96 (7.04–12.14)	0.170
PON1 (ng/mL)	12.93 ± 26.73 7.43 (6.26–9.49)	8.58 ± 2.83 7.80 (6.32–9.67)	7.92 ± 2.01 7.76 (5.76–9.05)	0.240 * for 0–2
PON2 (ng/mL)	73.04 ± 45.50 60.65 (35.16–116.72)	78.80 ± 52.00 70.53 (34.73–120.66)	82.68 ± 47.01 84.32 (38.58–117.37)	0.620
PON3 (pg/mL)	287.23 ± 945.302 93.54 (83.94–131.76)	104.07 ± 27.86 94.37 (84.43–114.32)	92.65 ± 21.67 85.12 (81.58–90.51)	0.172
AC (µM)	324.93 ± 79.81 339.82 (293.65–381.70)	337.64 ± 43.02 335.21 (309.44–380.157)	304.83 ± 99.46 326.26 (293.65–377.58)	0.042 * for 0–2
TOS (µM)	159.14 ± 38.94 168.00 (138.00–173.00)	148.14 ± 90.40 128.00 (83.00–203.00)	143.25 ± 74.74 128.00 (98.00–183.00)	0.461
CRP (mg/L)	5.30 ± 5.12 3.95 (0.61–10.00)	6.76 ± 5.84 5.14 (1.36–12.83)	8.62 ± 6.21 8.03 (1.93–15.03)	0.008 * for 0–2

* Statistically significant differences in post hoc pairwise comparisons between non-smoking women with PCOS (1) and non-smoking women without PCOS (0); non-smoking women with PCOS (1) and smoking women with PCOS (2), and between non-smoking women with PCOS (1) and smoking women without PCOS (2). Legend: PCOS—polycystic ovary syndrome; BMI—body mass index, HOMA-IR—homeostatic model assessment for insulin resistance; TyG index—triglycerides and glucose index; LDLR—low-density lipoprotein receptor; oxLDL—oxidized LDL; PCSK9—proprotein convertase subtilisin/kexin type 9, PON—paraoxonase, CRP—C-reactive protein, AC—antioxidant capacity, TOS—total antioxidant status.

**Table 2:** Studied parameters in entire group of PCOS women divided according to BMI and HOMA-IR value.

Variables	Women with PCOS			
	BMI < 25	BMI ≥ 25	HOMA-IR < 2.0	HOMA-IR ≥ 2.0
	*n* = 33	*n* = 27	*n* = 39	*n* = 21
Age (years)	25.00 ± 4.24 24.00 (21.00–28.00)	26.11 ± 3.24 26.00 (24.00–29.00)	25.10 ± 4.28 24.00 (22.00–27.00)	26.24 ± 2.74 27.00 (24.00–29.00)
BMI (kg/m^2^)	21.03 ± 1.95 20.82 (20.08–22.14)	32.36 ± 5.00 * 31.24 (28.55–36.21)	22.43 ± 3.67 21.09 (20.20–24.34)	33.00 ± 5.66 * 32.83 (30.46–37.18)
HOMA-IR	1.25 ± 0.55 1.19 (0.83–1.52)	3.05 ± 2.08 * 2.76 (1.79–3.83)	1.20 ± 0.39 1.19 (0.84–1.50)	3.66 ± 2.01 * 2.91 (2.60–3.87)
Cotinine (ng/mL)	12.54 ± 21.04 0.41 (0.41–16.65)	21.45 ± 26.18 0.65 (0.41–47.31)	15.19 ± 24.50 0.41 (0.41–34.02)	16.57 ± 25.69 0.41 (0.41–24.94)
Castelli index I	2.67 ± 0.46 2.69 (2.32–3.02)	3.97 ± 1.18 * 3.45 (2.82–4.41)	2.70 ± 0.51 2.69 (2.33–2.90)	4.05 ± 1.17 * 3.53 (3.08–4.89)
Castelli index II	1.24 ± 0.41 1.50 (1.13–1.64)	2.13 ± 0.81 * 1.97 (1.46–2.56)	1.44 ± 0.42 1.46 (1.13–1.64)	2.30 ± 0.80 * 2.13 (1.75–3.02)
TyG index	8.20 ± 0.41 8.08 (7.89–8.48)	8.90 ± 0.69 * 8.85 (8.33–9.43)	8.17 ± 0.42 8.09 (7.89–8.34)	9.08 ± 0.57 * 9.11 (8.56–9.43)
LDLR (ng/mL)	17.50 ± 8.45 16.60 (11.27–20.67)	18.15 ± 8.20 17.33 (12.20–22.93)	17.45 ± 8.19 15.33 (11.27–21.73)	18.43 ± 8.38 17.73 (12.20–20.67)
oxLDL (mU/mL)	35.86 ± 14.18 37.23 (21.81–48.19)	41.80 ± 10.90 42.92 (33.04–51.30)	36.77 ± 14.18 38.32 (21.81–50.09)	41.81 ± 10.15 42.92 (34.39–50.09)
Vitamin D (ng/mL)	27.46 ± 9.68 26.70 (20.20–30.90)	23.31 ± 9.30 21.50 (15.90–27.00)	27.57 ± 9.13 26.70 (20.90–33.90)	21.91 ± 9.74 * 18.60 (15.90–22.20)
PCSK9 (ng/mL)	7.85 ± 2.97 8.05 (5.79–10.04)	9.94 ± 3.47 * 9.15 (7.25–12.72)	7.94 ± 2.98 7.87 (5.79–10.09)	10.37 ± 3.49 * 9.33 (7.78–12.72)
PON1 (ng/mL)	8.31 ± 2.89 7.74 (6.20–9.23)	8.38 ± 2.25 7.80 (6.32–9.67)	7.76 ± 2.23 7.41 (6.09–8.72)	8.94 ± 2.34 * 9.05 (7.21–10.38)
PON2 (ng/mL)	67.35 ± 48.73 66.18 (21.7–104.4)	95.39 ± 49.71 * 86.80 (52.5–149.9)	77.99 ± 50.80 80.27 (31.2–117.4)	84.16 ± 51.67 73.93 (41.2–120.7)
PON3 (pg/mL)	111.87 ± 32.47 101.12 (834–136.6)	88.37 ± 10.06 * 86.43 (82.5–91.1)	104.65 ± 31.69 87.85 (82.1–121.4)	94.71 ± 15.69 90.05 (85.1–94.4)
AC (µM)	316.30 ± 83.44 318.88 (301.4–384.8)	340.30 ± 44.18 357.60 (313.7–377.6)	319.67 ± 79.26 332.78 (301.4–377.6)	341.82 ± 40.32 348.23 (313.7–381.2)
TOS (µM)	142.69 ± 82.79 123.00 (85.5–190.5)	151.33 ± 91.94 130.50 (88.0–213.0)	143.81 ± 84.31 123.00 (83.0–198.0)	151.42 ± 91.18 128.00 (93.00–208.00)
CRP (mg/L)	4.46 ± 4.73 2.21 (0.9–6.5)	11.17 ± 5.62 * 12.29 (7.0–16.3)	5.62 ± 5.59 2.88 (1.0–10.0)	11.02 ± 5.57 * 11.85 (6.2–16.5)

* *p* < 0.05 when compared to subgroup with BMI < 25.0 or with HOMA-IR < 2.0, respectively. Legend: PCOS—polycystic ovary syndrome; BMI—body mass index, HOMA-IR—homeostatic model assessment for insulin resistance; TyG index—triglycerides and glucose index; LDLR—low-density lipoprotein receptor; oxLDL—oxidized LDL; PCSK9—proprotein convertase subtilisin/kexin type 9, PON—paraoxonase, CRP—C-reactive protein, AC—antioxidant capacity, TOS—total antioxidant status.

**Table 3:** Effect of tobacco smoking and BMI on examined parameters in PCOS women.

Variables	Non-Smoking Women with PCOS		Smoking Women with PCOS	
	BMI < 25	BMI ≥ 25	BMI < 25	BMI ≥ 25
	*n* = 23	*n* = 15	*n* = 10	*n* = 12
Age (years)	24.17 ± 3.21 24.00 (21.00–27.00)	26.31 ± 3.16 26.00 (26.00–29.00)	27.40 ± 5.65 28.50 (21.00–31.00)	25.67 ± 3.37 24.00 (23.50–27.00)
BMI (kg/m^2^)	21.24 ± 1.81 20.86 (20.26–24.49)	31.81 ± 4.53 * 31.23 (27.86–34.37)	20.63 ± 2.23 20.88 (19.96–22.04)	32.86 ± 5.58 * 30.87 (29.22–37.91)
HOMA-IR	1.35 ± 0.62 1.26 (0.85–1.56)	3.07 ± 1.63 * 2.76 (2.00–3.85)	1.18 ± 0.58 1.09 (0.81–1.50)	2.99 ± 2.56 * 2.35 (1.55–3.26)
Cotinine (ng/mL)	0.49 ± 0.32 0.41 (0.41–0.41)	0.44 ± 0.12 0.41 (0.41–0.41)	40.26 ± 20.06 41.62 (19.42–57.01)	48.46 ± 16.65 56.75 (33.05–63.27)
Castelli index I	2.70 ± 0.46 2.74 (2.32–3.08)	3.68 ± 1.08 * 3.45 (2.82–4.30)	2.59 ± 0.48 2.51 (2.25–2.90)	3.93 ± 1.33 * 3.77 (2.83–4.65)
Castelli index II	1.46 ± 0.42 1.51 (1.15–1.82)	2.07 ± 0.70 * 1.97 (1.46–2.41)	1.34 ± 0.40 1.34 (1.00–1.58)	2.20 ± 0.95 * 1.90 (1.47–2.79)
TyG index	7.95 ± 0.36 7.83 (7.71–8.12)	8.52 ± 0.55 * 8.31 (8.16–8.81)	8.06 ± 0.31 8.01 (7.82–8.40)	8.67 ± 0.52 * 8.58 (8.21–9.16)
LDLR (ng/mL)	17.46 ± 7.10 16.60 (12.27–20.67)	17.96 ± 6.10 17.73 (12.20–22.93)	17.59 ± 11.43 15.83 (7.80–21.40)	18.39 ± 10.23 16.43 (10.97–23.10)
oxLDL (mU/mL)	34.36 ± 14.33 36.69 (21.81–45.08)	40.51 ± 10.80 39.13 (32.23–50.09)	39.30 ± 13.93 40.48 (28.30–51.84)	43.42 ± 1.27 46.03 (39.74–51.71)
Vitamin D (ng/mL)	28.63 ± 9.84 26.20 (20.70–36.90)	23.61 ± 11.40 18.60 (14.70–28.70)	25.00 ± 9.31 27.80 (15.00–33.90)	22.93 ± 6.23 22.70 (17.75–26.65)
PCSK9 (ng/mL)	7.78 ± 3.17 8.08 (5.58–10.31)	9.52 ± 3.85 8.05 (7.02–11.65)	8.00 ± 2.60 7.72 (5.80–9.33)	10.46 ± 3.01 10.22 (7.45–12.91)
PON1 (ng/mL)	8.59 ± 3.15 7.74 (6.20–9.48)	8.57 ± 2.47 7.80 (6.32–10.38)	7.65 ± 2.19 7.45 (5.63–8.41)	8.15 ± 2.01 8.16 (6.62–9.12)
PON2 (ng/mL)	61.35 ± 47.15 51.66 (21.22–93.02)	104.81 ± 51.47 * 113.22 (57.07–154.15)	81.43 ± 52.71 79.99 (38.58–109.07)	83.62 ± 46.86 86.38 (43.54–118.18)
PON3 (pg/mL)	114.20 ± 32.08 104.55 (85.18–137.68)	88.54 ± 7.90 * 88.35 (82.48–94.37)	105.91 ± 34.65 83.57 (81.64–135.48)	88.15 ± 12.64 85.30 (82.42–89.66)
AC (µM)	339.42 ± 40.47 332.61 (304.72–386.8)	335.28 ± 48.92 332.78 (313.73–377.07)	264.94 ± 127.17 306.52 (226.81–342.57)	349.70 ± 34.53 358.53 (322.14–379.38)
TOS (µM)	142.32 ± 79.84 125.00 (83.0–198.0)	157.29 ± 110.40 143.00 (78.0–218.0)	143.50 ± 93.47 113.00 (88.0–183.0)	143.00 ± 60.64 130.50 (118.0–193.0)
CRP (mg/L)	4.08 ± 4.45 2.21 (1.03–5.84)	10.76 ± 5.47 * 12.07 (7.00–15.68)	5.24 ± 5.42 3.42 (0.88–7.44)	11.68 ± 5.73 * 13.48 (6.17–16.52)

* *p* < 0.05 when compared to non-smoking subgroup with BMI < 25.0 or to smoking subgroup with BMI < 25.0. Legend: PCOS—polycystic ovary syndrome; BMI—body mass index, HOMA-IR—homeostatic model assessment for insulin resistance; TyG index—triglycerides and glucose index; LDLR—low-density lipoprotein receptor; oxLDL—oxidized LDL; PCSK9—proprotein convertase subtilisin/kexin type 9, PON—paraoxonase, CRP—C-reactive protein, AC—antioxidant capacity, TOS—total antioxidant status.

**Table 4:** Effect of tobacco smoking and HOMA-IR value on examined parameters in PCOS women.

Variables	Non-Smoking Women with PCOS		Smoking Women with PCOS	
	HOMA-IR < 2.0	HOMA-IR ≥ 2.0	HOMA-IR < 2.0	HOMA-IR ≥ 2.0
	*n* = 25	*n* = 13	*n* = 14	*n* = 8
Age (years)	24.12 ± 3.32 24.00 (22.00–26.00)	26.54 ± 2.88 * 27.00 (25.00–29.00)	27.70 ± 5.69 27.50 (23.00–34.00)	25.75 ± 2.60 25.50 (24.00–28.00)
BMI (kg/m^2^)	22.14 ± 2.74 21.10 (20.34–23.94)	32.20 ± 5.42 32.83 (29.49–34.45)	23.30 ± 4.95 21.57 (19.96–25.95)	34.32 ± 6.17 34.22 (30.48–39.56)
HOMA-IR	1.20 ± 0.38 1.17 (0.86–1.48)	3.53 ± 1.46 2.91 (2.76–3.87)	1.19 ± 0.43 1.22 (0.83–1.50)	3.87 ± 2.79 2.95 (2.41–3.68)
Cotinine (ng/mL)	0.49 ± 0.32 0.41 (0.41–0.41)	0.45 ± 0.13 0.41 (0.41–0.41)	40.64 ± 18.50 41.62 (24.00–56.75)	50.47 ± 18.64 61.22 (31.40–64.62)
Castelli index I	2.67 ± 0.45 2.67 (2.42–3.01)	3.88 ± 1.02 * 3.45 (3.08–4.30)	2.75 ± 0.62 2.75 (2.33–2.90)	4.32 ± 1.41 * 4.36 (3.12–5.48)
Castelli index II	1.44 ± 0.41 1.46 (1.15–1.81)	2.21 ± 0.65 * 2.12 (1.80–2.41)	1.45 ± 0.46 1.47 (1.13–1.58)	2.44 ± 1.04 * 2.33 (1.60–3.30)
TyG index	7.90 ± 0.29 7.83 (7.71–8.03)	8.70 ± 0.49 * 8.77 (8.21–8.85)	8.14 ± 0.39 8.13 (7.86–8.22)	8.84 ± 0.45 * 8.93 (8.36–9.23)
LDLR (ng/mL)	17.64 ± 6.98 16.60 (12.73–21.73)	17.68 ± 6.20 17.73 (12.20–2.67)	17.11 ± 10.28 14.23 (7.80–21.40)	19.64 ± 11.49 17.90 (11.23–24.40)
oxLDL (mU/mL)	35.06 ± 13.95 37.23 (21.81–44.13)	40.12 ± 11.56 38.59 (32.23–50.09)	39.82 ± 14.58 43.86 (26.54–51.84)	44.57 ± 7.15 44.20 (39.74–49.34)
Vitamin D (ng/mL)	28.58 ± 9.89 26.60 (21.90–35.60)	22.77 ± 11.31 * 18.60 (15.90–22.30)	25.78 ± 7.61 27.25 (23.20–32.90)	20.53 ± 9.95 18.40 (16.55–21.80)
PCSK9 (ng/mL)	7.39 ± 3.02 7.50 (5.09–9.07)	10.53 ± 3.58 * 9.36 (7.93–11.65)	8.91 ± 2.75 8.31 (7.04–11.10)	10.10 ± 3.56 9.14 (7.35–13.65)
PON1 (ng/mL)	8.28 ± 3.02 7.66 (6.20–8.78)	9.16 ± 2.55 9.47 (7.04–11.32)	7.55 ± 2.02 7.42 (5.71–8.63)	8.60 ± 2.08 8.40 (7.52–9.70)
PON2 (ng/mL)	68.58 ± 49.29 62.70 (23.12–93.02)	98.47 ± 55.38 113.22 (43.03–151.61)	96.08 ± 50.57 104.05 (80.00–139.33)	60.91 ± 37.04 63.09 (28.86–86.35)
PON3 (pg/mL)	110.01 ± 32.28 101.24 (84.43–126.47)	92.65 ± 12.59 90.98 (84.68–94.37)	94.35 ± 28.92 183.21 (81.40–89.26)	98.05 ± 20.29 89.53 (85.12–108.15)
AC (µM)	335.01 ± 46.46 330.03 (303.95–384.79)	342.10 ± 39.81 348.23 (318.23–373.46)	293.72 ± 13.41 339.31 (285.94–359.56)	340.92 ± 48.28 343.08 (299.31–382.53)
TOS (µM)	147.17 ± 84.28 133.00 (80.5–205.5)	150.08 ± 108.03 110.50 (70.5–205.5)	137.62 ± 87.43 123.00 (87.43–183.0)	153.71 ± 56.01 133.00 (118.0–218.0)
CRP (mg/L)	4.79 ± 4.92 2.70 (0.98–6.51)	11.04 ± 5.85 * 11.85 (7.00–15.86)	7.16 ± 6.60 6.75 (1.02–13.49)	10.98 ± 5.56 * 11.78 (5.62–16.51)

* *p* < 0.05 when compared to non-smoking or smoking subgroup with HOMA-IR < 2.0, respectively; BMI—body mass index, HOMA-IR—homeostatic model assessment for insulin resistance; TyG index—triglycerides and glucose index; LDLR—low-density lipoprotein receptor; oxLDL—oxidized LDL; PCSK9—proprotein convertase subtilisin/kexin type 9, PON—paraoxonase, CRP—C-reactive protein, AC—antioxidant capacity, TOS—total antioxidant status.

**Table 5:** Correlation coefficients between PCSK9, LDLR, oxLDL, TyG, vitamin D, PON1, PON2, or PON3 levels and other analyzed parameters in the whole group of women with PCOS.

Variables	PCSK9 (ng/mL)	LDLR (ng/mL)	oxLDL (mU/mL)	TyG Index	Vitamin D (ng/mL)	PON1 (ng/mL)
Age (years)	r = 0.28 *p* = 0.030	ns	ns	ns	ns	ns
PCSK9 (ng/mL)	-	r = 0.28 *p* = 0.029	r = 0.48 *p* < 0.001	r = 0.39 *p* = 0.002	ns	ns
LDLR (ng/mL)	r = 0.28 *p* = 0.029	-	ns	ns	ns	ns
oxLDL (mU/mL)	r = 0.48 *p* < 0.001		-	r = 0.29 *p* = 0.027	ns	ns
Vitamin D (ng/mL)	ns	ns	ns	ns	-	r = 0.27 *p* = 0.031
PON1 (ng/mL)	ns	ns	ns	r = 0.27 *p* = 0.038	ns	-
PON2 (ng/mL)	ns	ns	ns	ns	ns	ns
PON3 (pg/mL)	ns	ns	ns	ns	ns	r = 0.49 *p* < 0.001
CHO (mg/dL)	r = 0.52 *p* < 0.00	ns	r = 0.44 *p* < 0.00	r = 0.29 *p* = 0.026	ns	ns
HDL-C (mg/dL)	ns	ns	ns	ns	ns	r = −0.26 *p* = 0.04
LDL-C (mg/dL)	r = 0.49 *p* < 0.00	ns	r = 0.36 *p* < 0.00	r = 0.29 *p* = 0.025	ns	ns
Ty (mg/dL)	r = 0.48 *p* < 0.00	ns	r = 0.26 *p* < 0.05	r = 0.88 *p* < 0.001	ns	ns
Castelli index I	r = 0.43 *p* < 0.001	ns	r = 0.29 *p* = 0.030	r = 0.72 *p* < 0.001	ns	r = 0.30 *p* = 0.020
Castelli index II	r = 0.39 *p* < 0.003	ns	r = 0.30 *p* = 0.020	r = 0.612 *p* < 0.001	ns	r = 0.31 *p* = 0.015
Glucose 0′ (mg/dL)	r = 0.27 *p* < 0.05	ns	r = 0.25 *p* < 0.05	r = 0.41 *p* = 0.001	ns	ns
Glucose 120′ (mg/dL)	ns	ns	ns	r = 0.66 *p* < 0.001	ns	ns
Insulin 0′ (mU/mL)	ns	ns	ns	r = 0.65 *p* < 0.001	r = −0.29 *p* = 0.02	ns
TyG index	r = 0.39 *p* = 0.002	ns	r = 0.29 *p* = 0.027	-	ns	ns
HOMA-IR	ns	ns	ns	r = 0.65 *p* < 0.001	r = −0.26 *p* = 0.045	ns
AC (µM)	ns	ns	ns	ns	ns	ns
TOS (µM)	ns	ns	ns	ns	ns	r = 0.28 *p* = 0.042
CRP (mg/L)	r = 0.31 *p* = 0.019	ns	r = 0.29 *p* = 0.031	r = 0.44 *p* < 0.001	ns	r = 0.38 *p* = 0.004

Legend: PCOS—polycystic ovary syndrome; PCSK9—proprotein convertase subtilisin/kexin type 9; LDLR—low-density lipoprotein receptor; oxLDL—oxidized LDL; PON-1—paraoxonase 1; PON-2—paraoxonase 2; CHO—cholesterol; HDL-C—low-density lipoprotein; LDL-C—low-density lipoprotein; Ty—triglycerides; TyG index—triglycerides and glucose index; HOMA-IR—homeostatic model assessment for insulin resistance; AC—antioxidant capacity; TOS—total antioxidant status; CRP—C-reactive protein; ns – not significant.

**Table 6:** Correlation coefficients between PCSK9, LDLR, oxLDL, TyG, vitamin D, PON1, PON2, or PON3 levels and other analyzed parameters in smoking PCOS women.

Variables	PCSK9 (ng/mL)	LDLR (ng/mL)	oxLDL (mU/mL)	TyG Index	Vitamin D (ng/mL)	PON1 (ng/mL)
Age (years)	ns	ns	ns	ns	ns	ns
PCSK9 (ng/mL)	-	ns	ns	r = 0.48 *p* = 0.024	ns	ns
LDLR (ng/mL)	ns	-	ns	ns	ns	ns
oxLDL (mU/mL)	r = 0.55 *p* < 0.001	ns	-	ns	ns	r = −0.45 *p* < 0.042
Vitamin D (ng/mL)	ns	ns	ns	ns	-	ns
PON1 (ng/mL)	ns	ns	r = −0.45 *p* < 0.042	ns	ns	-
PON2 (ng/mL)	ns	ns	ns	ns	r = 0.63; *p* < 0.002	ns
CHO (mg/dL)	r = 0.66 *p* < 0.001	ns	r = 0.47 *p* < 0.029	r = 0.45 *p* = 0.038	ns	ns
HDL-C (mg/dL)	ns	ns	ns	r = −0.71 *p* < 0.001	ns	ns
LDL-C (mg/dL)	r = 0.68 *p* < 0.001	ns	r = 0.49 *p* < 0.00	ns	ns	ns
Ty (mg/dL)	ns	ns	ns	r = 0.98 *p* < 0.001	ns	ns
Castelli index I	r = 0.44 *p* < 0.041	ns	r = 0.29 *p* = 0.030	r = 0.88 *p* < 0.001	ns	ns
Castelli index II	r = 0.46 *p* = 0.030	ns	r = 0.30 *p* = 0.020	r = 0.74 *p* < 0.001	ns	ns
Glucose 0′ (mg/dL)	r = 0.46 *p* = 0.030	ns	r = 0.25 *p* < 0.05	ns	r = −0.51 *p* = 0.016	ns
Glucose 120′ (mg/dL)	ns	ns	ns	r = 0.48 *p* = 0.025	ns	ns
Insulin 0′ (mU/mL)	ns	ns	ns	r = 0.56 *p* = 0.007	ns	ns
TyG index	ns	ns	ns	-	ns	ns
HOMA-IR	ns	ns	ns	r = 0.56 *p* < 0.008	ns	ns
AC (µM)	ns	ns	ns	ns	ns	ns
TOS (µM)	ns	ns	ns	ns	ns	r = 0.49 *p* = 0.034
CRP (mg/L)	ns	r = 0.47 *p* = 0.031	r = 0.29 *p* = 0.031	ns	ns	ns

Legend: PCOS—polycystic ovary syndrome; PCSK9—proprotein convertase subtilisin/kexin type 9; LDLR—low-density lipoprotein receptor; oxLDL—oxidized LDL; PON-1—paraoxonase 1; PON-2—paraoxonase 2; CHO—cholesterol; HDL-C—low-density lipoprotein; LDL-C—low-density lipoprotein; Ty—triglycerides; TyG index—triglycerides and glucose index; HOMA-IR—homeostatic model assessment for insulin resistance; AC—antioxidant capacity; TOS—total antioxidant status; CRP—C-reactive protein; ns – not significant.

## Data Availability

The data presented in this study are available upon request from the corresponding author.

## Supplementary Materials

The following supporting information can be downloaded at: https://www.mdpi.com/article/10.3390/ijms27010331/s1.

## Abbreviations

The following abbreviations are used in this manuscript:

PCOS	polycystic ovary syndrome
PCOM	polycystic ovarian morphology
IR	insulin resistance
T2D	type 2 diabetes
CVD	cardiovascular disease
BMI	body mass index
HOMA-IR	homeostatic model assessment for insulin resistance
CHO	cholesterol
HDL-C	high density lipoprotein
LDL-C	low density lipoprotein
Ty	triglycerides
TyG index	the triglycerides and glucose index
LDLR	low-density lipoprotein receptor
oxLDL	oxidized LDL
PCSK9	proprotein convertase subtilisin/kexin type 9
PON	paraoxonase
PONs	paraoxonases
AC	antioxidant capacity
TOS	total antioxidant status
CRP	C-reactive protein
OS	oxidative stress
ROS	reactive oxygen species
SREBP2	sterol regulatory element-binding protein 2
HNF1α	hepatocyte nuclear factor-1 alpha
