# Supplementary material for: Metabolic Determinants of PCSK9 Regulation in Women with Polycystic Ovary Syndrome: The Role of Insulin Resistance, Obesity, and Tobacco Smoke Exposure
Source: Int J Mol Sci. 2025 Dec 28;27(1):331. doi: 10.3390/ijms27010331 (PMC12786223; doi:10.3390/ijms27010331)
Supplement: Supplementary file 1 [file ijms-27-00331-s001.zip › ijms-4025243-supplementary.pdf]

Additional correlation analyses were performed in the entire group of women with PCOS as well as in the subgroup of smoking women with PCOS. Associations were assessed between PCSK9, LDLR, oxLDL, the TyG index, vitamin D, PON1 concentrations, and selected sex hormones, sex hormone-binding globulin (SHBG), the free androgen index (FAI), glucose levels (fasting and post-oral glucose tolerance test), and insulin concentrations.

Table S1. The correlation coefficients between PCSK9, LDLR, oxLDL, TyG, vitamin D, PON1, PON2 levels and earlier published parameters in the whole group of women with PCOS.

**Variables**	PCSK9 (ng/ml)	LDLR (ng/ml)	oxLDL (mU/mL)	TyG index	Vitamin D (ng/ml)	PON1 (ng/ml)	PON2 (ng/ml)
Glucose 0′(mg/dL)	r=0.27 p<0.05	ns	r=0.25 p<0.05	r=0.41 p=0.001	ns	ns	ns
Glucose 120′(mg/dL)	ns	ns	ns	r=0.66 p<0.001	ns	ns	ns
Insulin 0′(mU/mL)	ns	ns	ns	r=0.65 p<0.001	r= -0.29 p=0.02	ns	ns
LH (IU/L)	ns	ns	r=0,31 p=0.01	r=0.26 p=0.044	ns	ns	ns
FSH (IU/L)	r= -0.26 p<0.05	ns	ns	ns	ns	ns	ns
SHBG (nmol/L)	ns	ns	ns	r= -0.58 p<0.001	ns	ns	r=-0.35 p=0.01
DHEA-S (µg/dL)	ns	ns	ns	ns	ns	ns	r=0.29 p=0.02
tTest (ng/mL)	ns	ns	ns	r=0.38 p=0.003	ns	ns	ns
fTest (pg/mL)	ns	ns	ns	r=0.39 p=0.002	ns	ns	ns
AD (ng/mL)	ns	ns	ns	ns	ns	ns	ns
FAI	ns	ns	ns	r=0.59 p<0.001	ns	ns	ns
AMH (ng/mL)	r=0.30 p=0.02	ns	ns	ns	ns	ns	r=-0.30 p=0.02

Legend: PCOS-polycystic ovary syndrome; PCSK9 - proprotein convertase subtilisin/kexin type 9; LDLR – low-density lipoprotein receptor; oxLDL – oxidized LDL; TyG index - the triglycerides and glucose index; PON-1 – paraoxonase 1; PON-2 – paraoxonase 2; LH-luteinizing hormone; FSH-follicle-stimulating hormone; SHBG-sex hormone-binding globulin; DHEA-S-dehydroepiandrosterone sulfate; tTest -total testosterone; fTest – free testosterone; AD – androstenedione; FAI – free androgen index; AMH – Anti-Müllerian hormone; 17-β-E2 -17 β -estradiol;17-OH P – 17-α-hydroxyprogesterone.

Table S2. The correlation coefficients between PCSK9, LDLR, oxLDL, vitamin d, PON1, PON2 or PON3 levels and other analyzed parameters in the smoking women with PCOS.

**Variables**	PCSK9 (ng/ml)	LDLR (ng/ml)	oxLDL (mU/mL)	TyG index	Vitamin D (ng/ml)	PON1 (ng/ml)	PON2 (ng/ml)
Glucose 0′(mg/dL)	r=0.46 p=0.030	ns	r=0.25 p<0.05	ns	r= -0.51 p=0.016	ns	ns
Glucose 120′(mg/dL)	ns	ns	ns	r=0.48 p=0.025	ns	ns	ns
Insulin 0′(mU/mL)	ns	ns	ns	r=0.56 p=0.007	ns	ns	ns
LH (IU/L)	ns	r=0.44 p<0.043	r=0,31 p=0.01	ns	ns	ns	ns
FSH (IU/L)	ns	ns	ns	ns	ns	ns	ns
SHBG (nmol/L)	ns	ns	ns	r= -0.62 p=0.002	ns	ns	r=-0.35 p=0.01
DHEA-S (μg/dL)	ns	ns	ns	ns	ns	ns	r=0.46 p=0.034
tTest (ng/mL)	ns	ns	ns	ns	ns	ns	ns
fTest (pg/mL)	ns	ns	ns	r=0.47 p=0.026	ns	ns	ns
AD (ng/mL)	ns	ns	ns	ns	ns	ns	ns
FAI	ns	ns	ns	r=0.60 p=0.003	ns	ns	ns
AMH (ng/mL)	r=0.43 p=0.044	ns	r=0.43 p=0.046	ns	ns	ns	ns

Legend: PCOS-polycystic ovary syndrome; PCSK9 - proprotein convertase subtilisin/kexin type 9; LDLR – low-density lipoprotein receptor; oxLDL – oxidized LDL; TyG index - the triglycerides and glucose index; PON-1 – paraoxonase 1; PON-2 – paraoxonase 2; LH-luteinizing hormone; FSH-follicle-stimulating hormone; SHBG-sex hormone-binding globulin; DHEA-S-dehydroepiandrosterone sulfate; tTest -total testosterone; fTest – free testosterone; AD – androstenedione; FAI – free androgen index; AMH – Anti-Müllerian hormone; 17-β-E2 -17 β -estradiol;17-OH P – 17-α-hydroxyprogesterone.
